# Factors Influencing the Executive Functions of Male and Female Cadets

**DOI:** 10.3390/ijerph192417043

**Published:** 2022-12-19

**Authors:** Grzegorz Zurek, Dariusz Lenart, Maciej Lachowicz, Krzysztof Zebrowski, Dariusz Jamro

**Affiliations:** 1 Department of Biostructure, Wroclaw University of Health and Sport Sciences, 51-612 Wroclaw, Poland; 2 Department of Physical Education and Sport, General Tadeusz Kosciuszko Military University of Land Forces, 51-147 Wroclaw, Poland

**Keywords:** executive functions, physical fitness, shooting performance, cadets, gender differences

## Abstract

Executive functions (EFs) are related to human abilities that allow individuals to achieve planned goals, contribute to creativity and the analysis of new ideas, and allow for adaptation to new situations in daily life. Thorough analyses of the factors affecting EFs can aid in the development of appropriate training programs for various social and professional groups, including the military. The purpose of this study was to determine the factors affecting the EFs of cadets (18 women and 108 men) studying at a military academy in Poland after the first and second terms of military training, and to investigate gender differences in the level of EFs, shooting performance (SP), and components of physical fitness (PF). The Neuropsychological Color Trails Test (CTT-2) was used to determine some of the EFs of the test subjects. Meanwhile, the level of SP was represented by the score achieved during marksmanship training implemented during military training. Assessment of the subjects’ PF was guided by the principles of the Health-Related Fitness assessment concept, i.e., health-related fitness. Differences between men and women in specific variables were calculated using the Mann–Whitney U test for independent samples, whilst the relationship between variables was analyzed using the best subset regression method. The results revealed that cadets’ EFs were influenced by their SP and their level of strength. However, there were no significant differences between male and female cadets in the levels of EFs or SP.

## 1. Introduction

Executive functions (EFs) are a set of control processes used when automatic action and reliance on instinct or intuition is imprudent, insufficient, or impossible [1]. According to the literature, EFs contribute to maintaining both cognitive and mental well-being [2], as well as maintaining a high level of physical fitness (PF) [3]. They also condition high performance in education [4] and professional careers [5], as well as when carrying out daily duties and tasks [6]. Furthermore, EFs ensure balanced social, psychological, and cognitive development [1]. Miyake et al. pointed to three main components of EF: cognitive set-shifting, performance monitoring, and response inhibition [7]. Cognitive set-shifting uses the short-term memory to allow switching between rules or mental sets that align with emerging requirements for the appropriate performance of a given task [8]. Performance monitoring is used to observe and correct one’s behavior while performing various tasks. With performance monitoring, the working memory can be updated to avoid making the same mistakes in the future [9]. Finally, response inhibition is associated with the withholding of behaviors that are inappropriate for a given task. Indeed, recognizing behaviors that are effective and important for success in performing an activity ultimately translates into direct action that achieves the desired results [10].

Research into EFs has focused on aspects that can have either a positive or negative effect on individuals [1]. Such research has been increasingly carried out in military personnel and has demonstrated poor EFs to be associated with obesity and less frequent physical activity [11]. Among the factors that have been found to have a negative impact on EFs in soldiers is chronic exposure to hypoxia [12], whilst combat stress has been shown to lead to a deterioration of the processes related to attention and to reduce the activity and integrity of the brain [13]. On the other hand, military training has been shown to contribute to the ability to maintain control over thoughts and emotions [14] and to improve overall EFs [15]. Indeed, physical activity is considered to be the main factor that brings about beneficial and significant improvements in EFs, such as increased information processing, reaction time, and decision-making, as well as improved attention and memory [16,17].

The study of EFs among soldiers, particularly cadets being trained as future commanders, should be of continuous interest to military researchers. An example of this is inhibitory control [18], which blocks behaviors and stops inappropriate automatic responses, ultimately changing the response to a better, more thoughtful, and situation-appropriate one [19]. This leads to improved effectiveness during tasks and increases the likelihood of success. In addition, military commanders often perform work under high-stress conditions, making life-or-death decisions, so they must develop appropriate action strategies and control their emotions.

Combat positions in military units pose great challenges, both physically and mentally, and such positions have been occupied by women for some time. For this reason, it is important to continuously study gender differences at various levels in military populations, which will bring about a greater understanding of the effects of military training and help to develop training plans. However, research on gender differences in the context of EFs has provided conflicting results. While some tasks have consistently revealed gender differences, for example, males perform significantly better in spatial tasks [20], others do not, such as the Wisconsin Card Sorting Test or the Stop Signal Task [21]. The literature has also highlighted differences between men and women regarding neuronal networks. Indeed, men and women use different neural strategies when performing the same cognitive tasks, showing different activities within specific areas of the brain that respond to specific EFs. These differences may be related to the observed gender differences in the structure, connectivity, and neurochemistry of the brain [22]. A holistic understanding of gender differences among soldiers can significantly improve the effectiveness of training and help to accurately understand the mechanisms responsible for gender behavioral differences.

Military combat readiness is primarily linked to soldiers sustaining their overall health and PF at a level that allows them to perform their required duties. Combat missions can last for an extended period of time, and soldiers, both men and women, are expected to maintain a high level of performance during this time. In regard to these increasing expectations, research is continuing on gender differences among soldiers across different levels. Research has highlighted differences in endurance, efficiency, and muscular fitness between male and female soldiers [23]; as a result of which, women are exposed to greater physiological stress during similar military activities [24]. Differences in PF between men and women are the subject of research among both cadets [25] and operational troops [23], and research has also focused on gender-specific adaptation to military training [26]. Meanwhile, women suffer injuries to the musculoskeletal system more often than men, which are the main cause of morbidity among soldiers [27].

The main issue of research in military populations comes down to the effectiveness of military training, which can be measured by the combat training of soldiers and the readiness of the army to defend the borders and national security. One of the most important components of a soldier’s combat training is their level of shooting performance (SP), so it is reasonable to research the differences between men and women in this respect. Such gender differences in SP have been analyzed during the World Cup sprint competition in biathlon [28] and Olympic rifle shooting [29], but no differences were found. However, the amount of research on these differences in military settings is quite scarce, or the findings are proprietary and unpublished due to their possible exploratory value to intelligence officers. In a study among soldiers, there were no statistically significant gender differences in two key SP parameters, including the proximity of shots to the center of the target and the proximity of shots to each other regardless of proximity to the center of the target [30]. In contrast, Johnson et al. showed that men and women differed in rifle shooting accuracy while on guard post duty, however, it is worth noting that these differences appeared after only 1.5 h of such duty [31]. Furthermore, the above studies were conducted using laser simulators. It seems reasonable to supplement the literature with studies evaluating shooting under range conditions and as close as possible to the battlefield.

In conclusion, research on the differences between female and male soldiers in such factors as PF, EFs, and combat training, provides invaluable information for commanders and training organizers. Furthermore, this research has allowed for continuous observation of the level of combat fitness of the army and can be used to direct training programs towards optimally bridging the greatest gender differences and maintaining soldiers in a state of high well-being. Moreover, it is also reasonable to analyze the various elements of military training that could potentially affect the EFs of cadets, future commanders, and leaders. Indeed, such individuals have the highest requirements for PF and cognitive fitness, as it is they who bear the greatest responsibility for the decisions made and tasks carried out for which a high level of EF is certainly required.

The main purpose of this research was to attempt to understand whether or not the variables analyzed can explain (and if so, which variables) the EFs of both male and female cadets after the first and second semesters of military training. In addition, the work aimed to examine whether significant differences exist between male and female cadets (and if so, what kind) at the level of individual components of PF and in EFs and SP.

In the research, it was hypothesized that the EFs of cadets will be determined by their SP and PF, however, it is difficult to predict exactly which components of PF to include in the EF prediction model. Indeed, the vast majority of studies to date have analyzed one-dimensional relationships between individual components of PF (mainly cardio-respiratory fitness) and the level of EFs [32], while the current research applied the multidimensional analysis of many components of PF and SP. The theoretical basis for making such a hypothesis was derived from the literature, in which EFs were positively related to shooting achievements (shooting with a pistol, a carbine, and shooting in a gas mask) [33], as well as with a higher PF [16,17]. Shooting training in its technical specificity, i.e., the shooter’s activities (focus on the target, shifting attention, controlling emotions, etc.) can be classified as specific training of EFs. It is also important to recognize the crucial role of attention in the complex process of shooting. In addition, subjects with a higher level of PF have better cognitive control and exhibit higher cognitive flexibility [34]. Considering that EFs are controlled by the anterior cingulate cortex, the basal ganglia, and the prefrontal cortex, it can be assumed that a high level of PF is beneficial to these neuronal regions [35]. It has also been assumed, based on the literature highlighted in the introduction, that male and female cadets will not differ in their level of EFs or SP [36,37].

## 2. Materials and Methods

The study was conducted among cadets of the Military University of Land Forces in Wroclaw (MULF) who had successfully passed the 1st and 2nd semesters of study. Cadets performed military service at the same time as they were studying. The cadets, after completing their 5-year training, will begin professional military service. MULF graduates have the appropriate knowledge and competencies needed to take command positions in military units.

The participants of the study gave written informed consent to participate in the project prior to the start of the study. In addition, the authors of the project fully informed the respondents about the objectives of the study, appropriate instructions were given, and appropriate questions were asked.

A total of 126 (18 females and 108 males) cadets met the inclusion criteria for the first and second phases of the study and were enrolled. The study was conducted at the MULF in January (Phase I of the study) and July (Phase II of the study) 2021.

### 2.1. Executive Functions

EFs and attention-related processes were examined using the Color Trails Test (CTT-2). The CTT-2 evaluates a variety of processes related to EFs, including maintaining and metastasizing attention, searching through material, monitoring one’s behavior, and the sequential processing of information. In addition, temporal indicators of CTT-2 performance are linked to the work of the brain’s frontal lobes. CTT-2 test sheets are printed in a 21.59 × 27.94 format on white paper. A single CTT-2 sheet consists of circles with numbers from 1 to 25 written in each circle. In addition, the circles are of different colors, yellow or pink. A significant complication is that each circle with a given number is printed twice in the above two colors. The CTT-2 neuropsychological test is used in studies among adults (18+) and its versatility lies in the fact that correct performance is not dependent on knowledge of any alphabet or language [37]. The test participant’s task is to connect the circles in ascending order from 1 to 25 as quickly as possible, while not taking the pencil off the page. The biggest difficulty is the condition that alternating colors must be maintained (e.g., yellow circle 1, pink circle 2, yellow circle 3, etc.). On the first page of the test sheet, the examinees performed a “warm-up” trial test. The main test was on the back of the sheet, which could not be turned over without the explicit instruction of the investigator. The test was performed on time with accuracy and without error. The execution time was counted in seconds. The stopwatch started when the test subject brought the pencil close to the first circle and was stopped when the test subject touched the last circle with the pencil. Task completion time was measured to 1 s. The CTT-2 test was conducted by a psychologist, and each time it took place in a lecture room, under the same conditions (psychologist—test subject) and at the same time of day.

### 2.2. Shooting Performance

The cadets’ SP was evaluated from tests carried out as part of their military studies and training program at the MULF. Within the framework of this study, the soldiers were mainly assessed on their performance under various types of shooting tasks using carbines, machine guns, and pistols. All tasks were undertaken in training ground conditions and at open combat ranges. All cadets had received the same amount of shooting training as part of the standardized shooting training for the commander major. Shooting practice was always held during the day and under similar weather conditions.

### 2.3. Physical Fitness

PF was assessed by conducting PF tests and taking measurements according to the health-related fitness (H-RF) concept [37].

#### 2.3.1. Anthropometric Measurements

The subjects’ body height (measurement accuracy of 1 mm) and body weight (measurement accuracy of 0.1 kg) were measured. Measurements were carried out using the SECA medical anthropometer and the Martin technique. In addition, body mass index (BMI) was calculated based on the somatic parameters. Additional data on the age and gender of the subjects were determined using a survey questionnaire.

#### 2.3.2. Motor Components

Motor components of PF were examined by measuring the dominant handgrip strength, speed (10 × 5 m shuttle run), endurance (1000 m run), lower limb explosive power (horizontal jump), and abdominal muscle strength (sit-ups in 30 s). Examination of these motor components was carried out in accordance with the guidelines of “Eurofit for adults” and the International Physical Fitness Test [37].

##### Dominant Handgrip Strength

The test of dominant hand strength was carried out in a sitting position, with the arms adducted, naturally rotated, maintaining a right angle at the elbow, and with the forearm positioned naturally. The test subject clenched the hand using maximum force. A Stanley hydraulic hand dynamometer was used for the test and measurements were accurate to 1 kg. In accordance with the recommendations of The American Society of Hand Therapists, the test was performed three times, with the highest result used for analysis.

##### Running Speed

The starting position involved the participant positioning themselves in front of the starting line. At the “START” signal, the participant’s task was to run 10 times over a 5-m distance between two parallel lines marked on the sports hall floor. While turning, the test participant had to cross the marked lines with both feet and was forbidden from running backward or using their hands for support. The test was conducted once.

##### Endurance

At the signal “READY”, the test subject stood behind the starting line in the starting position. At the signal “START”, the subjects ran at the fastest possible pace to the finish line of 2.5 laps of a 400-m athletic stadium. Time was measured to the nearest 1 s. The trial was conducted in good weather conditions.

##### Explosive Power of the Lower Extremities

The test subject stood behind a line, then from a simultaneous bounce with both feet performed a long-distance jump onto a special non-slip mat. The jump was measured in cm, the test subjects performed the test twice, and the result of the best jump was used for analysis. Any jump that resulted in a backward fall was invalid and the test was repeated.

##### Abdominal Muscle Strength

The test subject lay on a mattress with feet 30 cm apart and knees bent at right angles. Hands were intertwined at the nape of the neck. Test subjects were assisted by a partner, who held the feet so that they did not pull away from the ground. At the “START” signal, the subjects performed forward bends so that the elbows touched the knees, then returned to the starting position. The number of bends performed in 30 s was recorded. The test subject was not disqualified if he or she took longer breaks while performing the inclinations.

#### 2.3.3. Body Composition Measurements

Body fat and muscle mass were measured on a TANITA medical body composition analyzer using electrical impedance, also known as bioelectrical impedance analysis (BIA). The test subject stood straddling the marked fields (electrodes) with their hands straightened at the elbows and pointing away from the body, which allowed the current to flow freely through the body. Their hands touched electrodes attached to the special moving arms of the device.

The legal basis for conducting this research was the consent of the Rector—Commandant of the university (No. 271, dated 18 January 2021). In addition, the use of human subjects was consented to by the Senate Committee on Research Ethics of the Academy of Physical Education in Wroclaw (No. 2/2021, dated 12 February 2021).

### 2.4. Statistical Analysis

The results from the measurements of each variable were statistically analyzed. The distribution of data was assessed using the Kolmogorov–Smirnov test, and all variables analyzed had normal distributions. The Mann–Whitney U test for independent samples was used to examine variation in the mean values of the CTT-2 scores as well as the individual components of PF, and SP of participants.

The relationship between explanatory variables was analyzed using regression—the best subset method [38,39]. The explanatory variables in the regression analysis were the results of the CTT-2, while the explanatory variables were the components of PF (body height, body weight, BMI, body fat, muscle mass, dominant handgrip strength, horizontal jump, sit-ups, 10 × 5 m shuttle run, and 1000 m run) and the subjects’ SP.

Calculations were performed using professional Statistica v. 13 software (StatSoft, Tulsa, OK, USA) at the ISO 9001-certified Biostructure Research Laboratory of the University School of Sports and Health Sciences.

## 3. Results

Variations in the mean values of the variables tested by the Mann–Whitney U test in the first and second phases of the study are presented in Table 1 and Table 2. The analysis between men and women revealed statistically significant differences in all analyzed components in both the first and second phases of the study.

Men were characterized by a significantly higher body height, body weight, and muscle mass, higher BMI, and greater grip strength of the dominant hand. In addition, they had a longer horizontal jump, a shorter time in the 10 × 5 m shuttle run and the 1000 m endurance run, and significantly lower body fat. The men also performed a higher number of sit-up repetitions.

Of particular note are the results showing that there were no significant differences between men and women in the level of EFs and the level of SP during both the first and second phases of the study. Based on the arithmetic means, women achieved a shorter time in the CTT-2 compared to men during both phases. It is worth mentioning that during the first phase the difference was more than 7 s, while in the second phase the difference was less than 1 s. Analysis of the SP in the first phase indicated that men performed higher on average, however, after the semester-long training period, it was the women who performed slightly better (Table 1 and Table 2). It was also noticeable that both men and women averaged significantly higher shooting scores after the six-month training period. Nonetheless, the results were not statistically significant, however, there is a need for further study in this area in an attempt to confirm or refute the noted trends.

Results of the regression analysis (using the best subset method) from the second phase of the study, i.e., after one year of training at the MULF, are presented in Table 3. Of the variables analyzed, SP and dominant handgrip strength were qualified for the EF prediction models in both the female and male groups. Though, only SP was found to be a statistically significant variable. In the women’s group, the level of SP (β = −0.80) and the dominant hand grip (β = −0.256) were strong determinants and explained 53% of the variation in the level of EFs (R^2^ = 0.53). In contrast, in the male group, the level of SP (β = −0.55) and the dominant hand grip (β = −0.002) were found to be determinants of EFs and explained 29% of the variation (R^2^ = 0.29). In both models, the SP of the subjects was a statistically significant variable.

Regression analysis was also conducted during the first phase of the study, i.e., after a semester of military training at the MULF. Of the explanatory variables analyzed, no variable or group of variables from the study affected cadet EFs. A blank table was not attached to the aforementioned results due to the lack of variables in the model.

## 4. Discussion

The military combat readiness of a military unit depends to a large extent on a high level of health, including the PF of soldiers, which translates directly into the quality of their daily duties. Significant differences in PF according to HR-F between men and women are not surprising and do not raise doubts that men are generally physically fitter than women. These differences are linked to, among other things, the well-known sexual dimorphism. Sexual dimorphism is largely genetically determined and influences the bidirectional development of morphological and physiological, as well as psychological traits, in men and women according to sex [40]. Higher levels of strength in men are a result of, among other things, higher levels of testosterone. This hormone is indirectly responsible for greater muscle growth and development, especially during puberty in men. This was confirmed in the current study, in which men also had significantly higher levels of muscle mass.

The higher level of strength in men is also a result of differences in muscle structure between men and women. Specifically, men’s muscle tissue is composed mainly of type II fast-twitch muscle fibers; additionally, compared to women, men have a higher ratio of type II muscle fibers to type I. The greater level of muscle strength of men compared to women is also linked to their greater somatic development. Men in the current study surpassed women in body height and muscle mass [41,42]. The marked gender differences in body size and muscle mass in our study contribute not only to lower strength but also to lower power (anaerobic), as seen in the horizontal jump, compared to the women studied.

With research on military populations, the goal is to make the findings as practical as possible. One such practical result that will serve the command and teaching staff of the MULF is the significant gender difference in the level of strength of the cadets studied. As a result, the education and training plans should focus on bridging the differences in strength levels and on adjusting the loads for women accordingly. There is no doubt that strength is a major component of PF that determines the effective performance of difficult combat tasks, especially during training ground exercises such as marching and running with loads, operating in tactical gear, operating in urbanized terrain, transporting the wounded, and logistics transport, etc. Field exercises, as well as potential future warfare, require heavy and continuous use of force. Women, compared to men, have significantly limited capabilities under such conditions. In addition, studies report that as a result of performing tasks that require the use of force, women are much more likely to suffer numerous injuries and strains to, for example, the knee joints and lumbar spine [23,42].

Numerous studies at other military academies around the world, as well as in other military units, have addressed the topic of PF and its potential links to other factors, and have highlighted its value in comparative analysis. An integral component analyzed in these studies is the strength level of cadets [25,43]. Strength is considered one of the determinants of the ability to lift and carry weights manually—an ability desired in military service. In a study by Leyk et al., the maximum isometric hand strength was determined in 1654 healthy men and 533 women aged 20–25 years. The mean maximum hand strength showed, as in the study among MULF cadets, the expected marked difference between men (541 N) and women (329 N) [44]. In a study by Mansour et al. among students, statistically significant differences in hand strength were also obtained, and the expected morphological differences (height, body weight, and body fat content) were also shown to be similar to the current study [45].

Results from studies at other military academic centers around the world have reported similar gender differences among cadets. A study of 2753 cadets (1046 women and 1707 men) from the U.S. Air Force, the Military Academy, and the U.S. Naval Academy found that men and women differed significantly in all strength tests, including handgrip strength and somatic variables (height, weight, and BMI) [46]. In contrast, in a study by Vikmoen et al. carried out during initial fitness measurements before demanding selection exercises, as in our study, women had a significantly lower weight and muscle mass compared to men, while men were characterized by lower levels of fat tissue compared to women. In the above studies, men also performed significantly better in tests such as the countermovement jump, evacuation test, and medicine ball throw [24]. It should be noted that these were different tests than those used in our study, but they certainly provide great comparative value.

The well-known sexual dimorphism is also clearly seen in the endurance abilities of men and women. In our study, men and women differed significantly in running the 1000 m distance during both the first and second phases of training. These differences explain the well-known physiological and genetic differences in the endurance abilities of men and women. According to the literature, women present, on average, a 10% lower level of peak oxygen uptake (VO2) per total body weight compared to men at similar levels of training and fitness [47], as well as having an inherently lower blood oxygen content [48]. In addition, the structural elements of the aerobic energy system that are key to performance are gender specific. Women generally have smaller hearts compared to men, even when normalized by body weight. The total amount of fluid filling the circulatory system, or blood volume, and the oxygen-carrying capacity of the blood are also lower in women [49]. In conclusion, men have a better ability to deliver oxygen to skeletal muscles, which is a major determinant of their higher endurance capacity.

Comparing the results of our study with other studies, similar differences were found between male and female cadets in endurance. In a study among British Army recruits both before and after undergoing basic military training, similar to our study, significant differences were found in aerobic capacity. Men achieved significantly better running times over a distance of 2.4 km [50]. Similar tests and measurements from our research were conducted in the Israel Defense Forces on a group of 257 willing soldiers, 199 of whom were women. The components were measured before the start of the 4-month integrated basic training program. Gender differences were observed not only in the 2 km running time, but also in lean mass, fat mass, body fat percentage, and maximum oxygen uptake (VO2 max). Men achieved a significantly better time in the running test and had a significantly higher VO2 max and higher lean mass, while they were characterized by a lower fat mass and lower body fat percentage. The above results corroborate reports from our research on gender differences in the morphological and muscular components of PF, including studies in which a significantly higher percentage were women [51].

Another study in the Israel Defense Forces was conducted by Yanovich et al. in an atypical military unit where 70% of the total serving soldiers were women. The study included 129 women and 47 men. Somatic measurements, as in our study, revealed the expected differences. Men were characterized by a higher body height (difference of 6.9%) and higher body weight (difference of 12.8%); in addition, men had a 0.9% lower BMI, and their percentage of fat tissue was 64.4% lower than women. The motor fitness tests also included tests of performing the maximum amount of muscular collapse during squats, push-ups, and forward trunk bends. Only the abdominal strength test showed no statistically significant difference, which is in contrast to our study. The tests of sit-ups from a lying position, however, differed significantly from our study. In Yanovich’s study, the test was performed until the muscle collapsed or until the subject paused for more than 2 s, while in the current study the subject was asked to perform sit-ups from lying down for 30 s [52].

EFs make it possible to create and analyze new ideas, and to take time to think before acting. It is through the use of EFs that a person can cope with new, unforeseen challenges and maintain concentration for a longer period of time. Although the research on EFs is quite rich in the literature, the impact of gender on EFs is still poorly studied and poorly characterized. In addition, there are conflicting results when it comes to gender differences in EFs. The results of our study showed that female and male cadets did not differ in the level of EFs. Previous studies have revealed that women were more prone to interfering with task-irrelevant information compared to men [53,54] while other studies did not show this [55]. Another study found differences at the neuronal level; however, no gender differences were found at the behavioral level where a Simon task was used [56].

A valuable systematic literature review of functional neuroimaging studies examining gender differences in three important domains of EFs, including performance monitoring, cognitive set-shifting, and response inhibition was conducted by Gaillard et al. The review included 21 studies with a total of 677 women and 686 men. The authors concluded that it is currently impossible to clearly define the exact differences between men and women in terms of EFs. The basis for such a statement is the fact that studies often use high methodological variability, moreover, they are dealing with the involvement of multiple neuronal networks. However, the literature has confirmed differences in neuronal networks between men and women. Indeed, women and men use different neuronal strategies when performing the same cognitive tasks [22].

Research on the effect of gender on EFs (sustained and metastatic attention, intentional searching of material, monitoring of one’s behavior, and sequential processing of information) using CTT has provided consistent results. Such research was conducted by Messinis et al. among others. The CTT neuropsychological test was applied to 163 healthy adult participants and showed a significant effect of age and education level on CTT completion time (a lower age and higher education level of the subjects positively influenced the results achieved in CTT). Meanwhile, in agreement with our study, gender did not affect completion time [57]. Similar results were found by Rabelo et al., who evaluated the CTT in a sample of healthy Brazilian individuals (1942 participants aged 18–86). The results of the study revealed that regardless of gender, CTT completion time worsened with increasing age of the subjects, and that completion time decreased (improved) with increasing educational level. In addition, as in our study, women and men did not differ in CTT completion time [58]. Overall, according to the above results and the CTT application manual, gender does not appear to have a significant role in CTT performance [36]. Based on our study and the available literature, we conclude that men and women do not show differences in EF levels when the tests are conducted using CTT in a group (as in our study) of similar age and educational level. To the best of our knowledge, to date, no studies have been conducted on gender differences in EFs in military populations or military academy training cadets in particular.

Another important finding from the present study is the lack of differences between male and female cadets in SP. Looking for a potential explanation for this result, it should be noted that shooting does not require as much physical effort compared to other sports [29]. Thus, skills such as accuracy and coordination of movements [59] and the level of EFs [32] may be key factors in shooting. According to the literature, gender differences in SP were found by Luchsinger et al., who conducted a study during a World Cup sprint competition in biathlon [28], and Goldschmied et al., who analyzed the shooting results of men and women in Olympic rifle shooting [29]. Thus, it can be concluded that men and women do not differ in SP when using military carbines and pistols, but they do when using biathlon and Olympic air rifles.

Another study providing comparative value with our research was conducted by Mon-López et al. who analyzed the shooting results from the World Shooting Championships among 704 athletes (349 men and 355 women). The main finding reported in the study was that women performed worse in small arms shooting, while no differences in performance were found in the long gun competitions. An overall analysis of all shooting, regardless of weapon type or shooting competition, was also performed and found no gender differences [60]. Thus, the results of our research are in agreement with the above results. However, it should be noted that there were significant differences in the level of SP between cadets who had a year of military training and highly trained athletes who were performing at the world championships. Shooting conditions and the type of weapons used are also important determinants of differences found. It is also worth noting that in terms of training soldiers, the ability to shoot carbines, more than other weapons, is essential. In potential front-line operations, soldiers performing tactical actions in organized teams use carbines during direct fire contact.

Studies in other uniformed formations have shown that men outperformed women in pistol shooting using various calibers. Different results were presented by Vučković et al., where men and women differed in SP, but only at the beginning of pistol training; importantly, these differences were equalized following the implementation of training. It was hypothesized that these differences may have been caused by differences in the strength of the finger flexor and shoulder adductor muscles necessary for stabilizing the pistol [61,62]. In the military community, there were also no perceived gender differences in long-gun shooting. In a study by Kemnitz et al. (2001) using the NOPTEL ST-1000 laser simulator, men and women shot equally accurately and precisely. The above research shows that men and women do not differ in rifle shooting when using either simulators or under field conditions similar to the current study [63]. Results presented in a study by Johnson showed that men and women differed in rifle shooting accuracy while on guard post duty. However, it is worth noting that these differences appeared only after 1.5 h of such duty. The authors attributed these differences to the lower strength and shoulder girth among women. Additionally, this study used a simulator designed for marksmanship training, and there is still a lack of studies in the literature that use real-world field or combat conditions to test soldiers’ marksmanship [31].

The main result of the present study is the regression analysis which demonstrated that the level of SP (β = −0.80) and hand grip strength (β = −0.256) are strong determinants of CTT-2 scores in females. Indeed, SP was a statistically significant variable in both models and explained 53% of the variation in the level of EFs in the females and 29% in the male subjects. To the best of our knowledge, the literature analyzing the influence of various factors on the level of soldiers’ EFs is quite poor. It can be assumed that the current study is likely the first to attempt to answer the questions posed in the hypothesis about whether there are variables among the components of SP that influence the level of cadets’ EFs. Associations between shooting fitness and EFs were found by Jamro et al. and indicated that the higher SP of male cadets in various shooting tasks (carbine shooting, pistol shooting, and gas mask shooting) and female cadets in pistol shooting were significantly positively correlated with higher EFs [64]. Since EFs are a very important factor for cadets’ combat training, it is also reasonable to look for factors among soldiers that significantly affect their EFs.

Of all the components of SP analyzed, only the strength of the dominant hand combined with SP entered the predictive model of EFs. Indeed, in both the male and female groups, the R^2^ coefficient of determination and standardized coefficients β were significantly higher in the female group, while SP was statistically significant. Among other factors, this result can be explained by the fact that EFs increase among cadets as their level of SP increases [64]. Furthermore, it is well known that by using specialized cognitive training and other methods we can improve and train EFs. Such functional training methods include CogMed computerized training, mindfulness, mixed martial arts, and general physical activity. The key is repeated practice and exercise, and constantly challenging the EFs in new ways. Regular mental exercise improves mental health, exactly as physical activity is one of the main conditions for maintaining a healthy body and overall well-being [1].

All of the conditions and factors that potentially improve EFs are fulfilled by SP, meaning that SP can be considered to be extended training of EFs. Indeed, while shooting, the shooter must focus intensively on shooting actions (pulling the trigger tongue, breathing, aiming, etc.). Concurrently, they must disregard irrelevant external factors such as noise. Moreover, when shooting, it is very important to maintain a high degree of self-control, consisting, among other things, of controlling emotions and refraining from impulsive action. In addition, dynamic shooting requires a very high degree of flexibility and rapid adaptation to new situations, often under time pressure [65]. All of the above technical components of shooting are part of the activities for which highly developed EFs are needed and explain the finding that the level of SP had a positive impact on the level of EFs in cadets.

The effect of SP on soldiers’ EFs can also be explained by coordination training, which has been confirmed in other studies to be associated with improved cognitive performance [66]. During shooting, soldiers must coordinate many specific activities simultaneously. For example, when shooting in a standing stance, the shooter must aim, work the trigger tongue, and maintain balance at the same time, while trying to move the weapon as little as possible. Even a slight movement of the barrel before firing at a long distance can cause a soldier to miss the target.

The results of the current study in the area of the effects of military training on the EFs of soldiers coincide with the results of a highly valuable study by Batouli and Saba. The latter used functional magnetic resonance imaging to examine the brain activity of Iranian army officers and a control group during a simulated aggressive and directly life-threatening situation. The study participants were graduates of the Iranian army’s 3-year, highly difficult, and both physically and mentally demanding basic training. The results of the study revealed a greater volume in 1103 brain voxels of military officers in as many as five brain areas. In addition, higher activation of the brain within a pattern processing area was observed in the military group compared to the civilian group. Thus, there is no doubt that military training affects positive structural changes in the brain. Moreover, the study showed significantly higher mental toughness of military officers in response to stressful situations compared to the control group [15]. The above results, combined with the results of our research, unquestionably support the beneficial effect of specialized military training on the EFs of soldiers. However, the aforementioned study did not indicate which exact elements of military training improved specific EFs. An important difference between the above research and our own is that the cadets in our study had one year of military training, while the Iranian army officers had three years of intensive training. Thus, it can be assumed that the impact of the SP analyzed in the current study may also have even greater benefits for the cadets’ EFs after three years of training. Thus, there is a need for additional research to better understand the relationship between these variables at different stages of military training.

The appearance of strength in the cadet EFs prediction model is explained by the fact that, as we know from the literature, generally higher levels of strength are associated with maintaining cognitive abilities at a high level [67]. In addition, resistance training contributes to improving the functional plasticity of response inhibition processes in the cerebral cortex, which is complementary to the aerobic training addressed in the study [68]. Interpretation of the influence of factors, such as strength and SP on women’s EFs compared to men, according to the literature, indicates that women show greater improvement in a number of variables using the same influence factor. For example, women show greater improvement compared to men after undergoing the same basic training program [69], and significantly improve their SP compared to men [61].

Summarizing reports from the literature and our research on gender differences in the PF of soldiers, further research should be conducted in this area, whilst also attempting to make the results practical. On the other hand, women and men serving in the military do not show significant differences in SP, so in this area, it is reasonable to conduct further SP without considering gender. The same situation applies to the level of EFs, in which, according to the results of our research as well as the available literature, women and men do not show differences. However, what is most important from the present study is that the level of EFs of the cadets studied is mainly influenced by SP and strength level. Moreover, the search for factors influencing soldiers’ EFs is particularly important because interventions that achieve even a small improvement can result in a large improvement in health and in task efficiency that, on an army-wide scale, can certainly raise the level of national security [70].

The results of the study confirm the hypothesis that cadets’ EFs will be influenced by their SP and PF. Specifically, the EF prediction model includes, in addition to the level of SP, the level of strength of the subjects, which was difficult to predict when formulating the hypothesis. It also confirmed our assumption that male and female cadets would not differ significantly in their level of EFs or SP.

## 5. Conclusions

Usually, during enlistment, men and women are assigned to the same structures and receive the same military training. Given the differences between men and women in PF, it is reasonable to use gender-specific training to bridge such differences and optimize training adaptations. On the other hand, in the area of SP and cognitive training (if any), it is reasonable to carry out further training without taking gender into account.

It is suggested that the main emphasis should be placed on marksmanship training and strength development in cadet training programs. Focusing on these elements of training will best equip future commanders to meet growing demands in the area of PF components, particularly in the area of cognitive fitness. We also suggest that military training should take into account the level of PF as well as the cognitive aspects that are crucial in the military service of commanders.

## 6. Limitations, Strengths, and Future Research

Significant differences and influences among the analyzed variables were shown in a sample with a small number of women relative to men. It is therefore reasonable to continue and repeat the study on a group with a similar percentage of women relative to men. We point out, however, that all men and women studying in the 1st year at the MULF participated in our research.

The level of EFs in our study was assessed using the CTT-2, but we suggest that other neuropsychological tests (e.g., stop signal task and Wisconsin card sorting test) should also be used in future studies. This will bring a greater understanding of the differences between men and women and the relationship between the variables studied and EFs [9,71].

Since we know from the present study that the predictive model of cadets’ EFs after one year of military training includes strength and marksmanship training, it would behoove subsequent studies to carefully examine if the benefits are sustainable, how long they last, and what intensity of use of these variables in military training is best? Additionally, will similar impacts also be observed in subsequent years of training?

## Figures and Tables

**Table 1:** Variation in the mean values of the somatic variables, PF, SP, and CTT-2 by the Mann–Whitney U test for independent samples between men and women—Phase I of the study.

Variable	Men (N = 108)			Women (N = 18)			Mann–Whitney U Test
	$\bar{x}$	sd	v	$\bar{x}$	sd	v	*p*
Body height (cm)	179.44	5.60	3.12	167.49	4.24	2.53	**0.0000**
Body weight (kg)	77.35	7.70	9.95	63.11	3.77	5.97	**0.0000**
BMI (kg/m^2^)	24.01	2.05	8.55	22.35	1.24	5.55	**0.0006**
Fat tissue (%)	13.92	3.14	22.54	22.42	3.22	14.38	**0.0000**
Muscle mass (kg)	62.36	5.35	8.58	45.03	2.31	5.12	**0.0000**
Dominant handgrip strength (kg)	124.48	19.14	15.38	87.50	14.17	16.19	**0.0000**
Horizontal jump (cm)	226.58	19.20	8.47	187.00	17.04	9.11	**0.0000**
Sit-ups (amount)	31.76	3.71	11.67	28.06	3.11	11.10	**0.0001**
Shuttle run 10 × 5 m (s)	18.47	1.01	5.48	19.84	0.82	4.12	**0.0000**
Run 1000 m (s)	213.91	23.69	11.07	245.44	14.26	5.81	**0.0000**
CTT-2 (s)	67.65	16.23	23.99	60.17	12.01	19.96	0.0646
SP (mark)	3.54	1.13	31.97	3.11	1.02	32.87	0.1389

**Table 2:** Variation in the mean values of the somatic variables, PF, SP, and CTT-2 as assessed by the Mann–Whitney U test for independent samples between men and women—Phase II of the study.

Variable	Men (N = 108)			Women (N = 18)			Mann–Whitney U Test
	$\bar{x}$	sd	v	$\bar{x}$	sd	v	*p*
Body height (cm)	179.46	5.64	3.14	167.43	4.46	2.67	**0.0000**
Body weight (kg)	76.63	7.89	10.29	61.96	3.47	5.59	**0.0000**
BMI (kg/m^2^)	23.78	2.14	9.01	22.12	1.28	5.78	**0.0008**
Fat tissue (%)	13.58	3.06	22.50	22.03	3.12	14.17	**0.0000**
Muscle mass (kg)	62.13	5.29	8.51	44.85	1.88	4.18	**0.0000**
Dominant handgrip strength (kg)	131.16	18.59	14.17	93.33	10.71	11.48	**0.0000**
Horizontal jump (cm)	228.86	18.95	8.28	186.83	19.92	10.66	**0.0000**
Sit-ups (amount)	30.49	4.12	13.51	27.94	3.73	13.36	**0.0228**
Shuttle run 10 × 5 m (s)	17.95	1.22	6.78	19.22	1.41	7.34	**0.0001**
Run 1000 m (s)	209.37	22.89	10.93	239.94	22.71	9.46	**0.0000**
CTT-2 (s)	59.22	12.16	20.54	58.67	11.43	19.48	0.9555
SP (mark)	4.11	0.60	14.56	4.39	0.41	9.42	0.0570

**Table 3:** Results of regression analysis (using the best subset method) of the level of EFs as a function of SP and PF—Phase II of the study.

Test for Full Model					Standardized Coefficients β at Selected Variables	
Variable	Gender	F	*p*	Adjusted R^2^	Dominant Handgrip Strength	SP
CTT-2	m	**23.11**	**0.000000**	**0.29**	−0.002439	**−0.55**
	w	**10.39**	**0.001468**	**0.53**	−0.256538	**−0.80**

Adjusted R^2^—coefficient of determination, β significant at the level of *p* < 0.05—marked in bold.

## Data Availability

The datasets used and/or analyzed during this study are available from the corresponding author upon reasonable request.
